# Clonal hematopoiesis and cardiovascular disease: deciphering interconnections

**DOI:** 10.1007/s00395-022-00969-w

**Published:** 2022-11-10

**Authors:** Anna Stein, Klaus Metzeler, Anne Sophie Kubasch, Karl-Philipp Rommel, Steffen Desch, Petra Buettner, Maciej Rosolowski, Michael Cross, Uwe Platzbecker, Holger Thiele

**Affiliations:** 1 Medical Clinic and Policlinic 1, Hematology, Cellular Therapy and Hemostaseology, Leipzig University Hospital, Liebigstr. 20, 04103 Leipzig, Germany; 2 Department of Internal Medicine/Cardiology, Heart Center Leipzig at the University of Leipzig and Leipzig Heart Institute, Strümpellstr. 39, 04289 Leipzig, Germany; 3 Institute for Medical Informatics, Statistics and Epidemiology, Medical Faculty, Leipzig University, Leipzig, Germany

**Keywords:** Clonal hematopoiesis, CHIP, Clonal hematopoiesis of indeterminate potential, Atherosclerosis, Heart failure, TET2, DNMT3A, JAK2

## Abstract

Cardiovascular and oncological diseases represent the global major causes of death. For both, a novel and far-reaching risk factor has been identified: clonal hematopoiesis (CH). CH is defined as clonal expansion of peripheral blood cells on the basis of somatic mutations, without overt hematological malignancy. The most commonly affected genes are *TET2*, *DNMT3A*, *ASXL1* and *JAK2*. By the age of 70, at least 20–50% of all individuals carry a CH clone, conveying a striking clinical impact by increasing all-cause mortality by 40%. This is due predominantly to a nearly two-fold increase of cardiovascular risk, but also to an elevated risk of malignant transformation. Individuals with CH show not only increased risk for, but also worse outcomes after arteriosclerotic events, such as stroke or myocardial infarction, decompensated heart failure and cardiogenic shock. Elevated cytokine levels, dysfunctional macrophage activity and activation of the inflammasome suggest that a vicious cycle of chronic inflammation and clonal expansion represents the major functional link. Despite the apparently high impact of this entity, awareness, functional understanding and especially clinical implications still require further research. This review provides an overview of the current knowledge of CH and its relation to cardiovascular and hematological diseases. It focuses on the basic functional mechanisms in the interplay between atherosclerosis, inflammation and CH, identifies issues for further research and considers potential clinical implications.

## Introduction

Hypertension, dyslipidemia, diabetes, obesity and smoking are the most common and widely known cardiovascular risk factors. Nevertheless, the global profile of atherosclerosis has changed substantially over the last decade. Given the association of cardiovascular diseases (CVD) with prosperity, worldwide socioeconomic development has spurred the spread of atherosclerosis around the globe. In parallel with the changes in distribution, risk stratification is also undergoing a transformation. Due to the effective and widely affordable administration of cholesterol reducing and anti-hypertensive drugs, risk factors beyond the traditional candidates are gaining impact. Consequentially, this has led to a shift in patient’s risk characteristics (e.g., toward younger age, female sex, normal body mass index [BMI] and different ethnicity). This emphasizes the need to understand atherosclerotic risk beyond metabolic syndrome and other traditional elicitors to prevent disease development in the future [59]. A novel, recently described non-traditional risk factor is a hematopoietic manifestation called CH.

In the broad sense, CH describes any clonal hematopoietic proliferation, including leukemias and other malignant hematological diseases. More narrowly, the term is used for clonal proliferation of hematopoietic cells without overt hematological malignancy. This definition of CH compromises two distinct, major entities: clonal hematopoiesis of indeterminate potential (CHIP) and clonal cytopenia of undetermined significance (CCUS), which is distinguished from CHIP by the presence of cytopenias.

CHIP was first recognized in the 1990s, when Fey et al. observed skewed X-linked inactivation in older women and were the first to propose a potential mechanism of acquired clonality. Later, the same group identified specific somatic mutations in some of the affected women [23, 38] and referred to the condition as being “of indeterminate potential” because of the lack of a clear association to disease. Since then, however, research has provided mounting evidence to the contrary.

During aging, proliferating cells acquire somatic mutations due to stress, exogenous factors and increasing susceptibility to errors during DNA-replication. Most of these mutations are either neutral or detrimental at the single cell level and remain insignificant. Others, if occurring within a particular driver gene or regulatory sequence, may bestow a selective advantage and even contribute to malignant transformation. Along this evolutionary process, transitions are fluid and pre-malignant lesions are common for most oncologic entities. Recognition of this has identified valuable opportunities for screening approaches and pre-emptive treatment before disease development. However, contrary to other pre-malignant entities, CHIP conveys much broader systemic impact not limited to the hematopoietic system. In particular, it has been shown to be associated with increased all-cause mortality and cardiovascular risk [54, 55].

Given the clinical significance as a pre-malignant state and cardiovascular risk factor, CHIP is gaining increasing recognition as a distinct and relevant clinical entity. This is leading to discussions about screening and precautionary interventions that draw a unique and promising connection between the hemato-oncological and cardiovascular fields in terms of current and future (preventive) clinical approaches. However, the importance of this phenomenon has not gained enough awareness, yet. A concerted basic and translational research effort will be required to establish standard clinical guidance recommendations. Not least, several crucial questions remain unanswered: How to screen? Who to screen? Who to treat and how to treat?

## Definition of CHIP: how to measure?

CHIP is historically defined as the presence of somatic mutations in the peripheral blood with a clonal size of at least 2% variant allele frequency (VAF), in the absence of overt hematological disease [84].

**Fig. 1:**
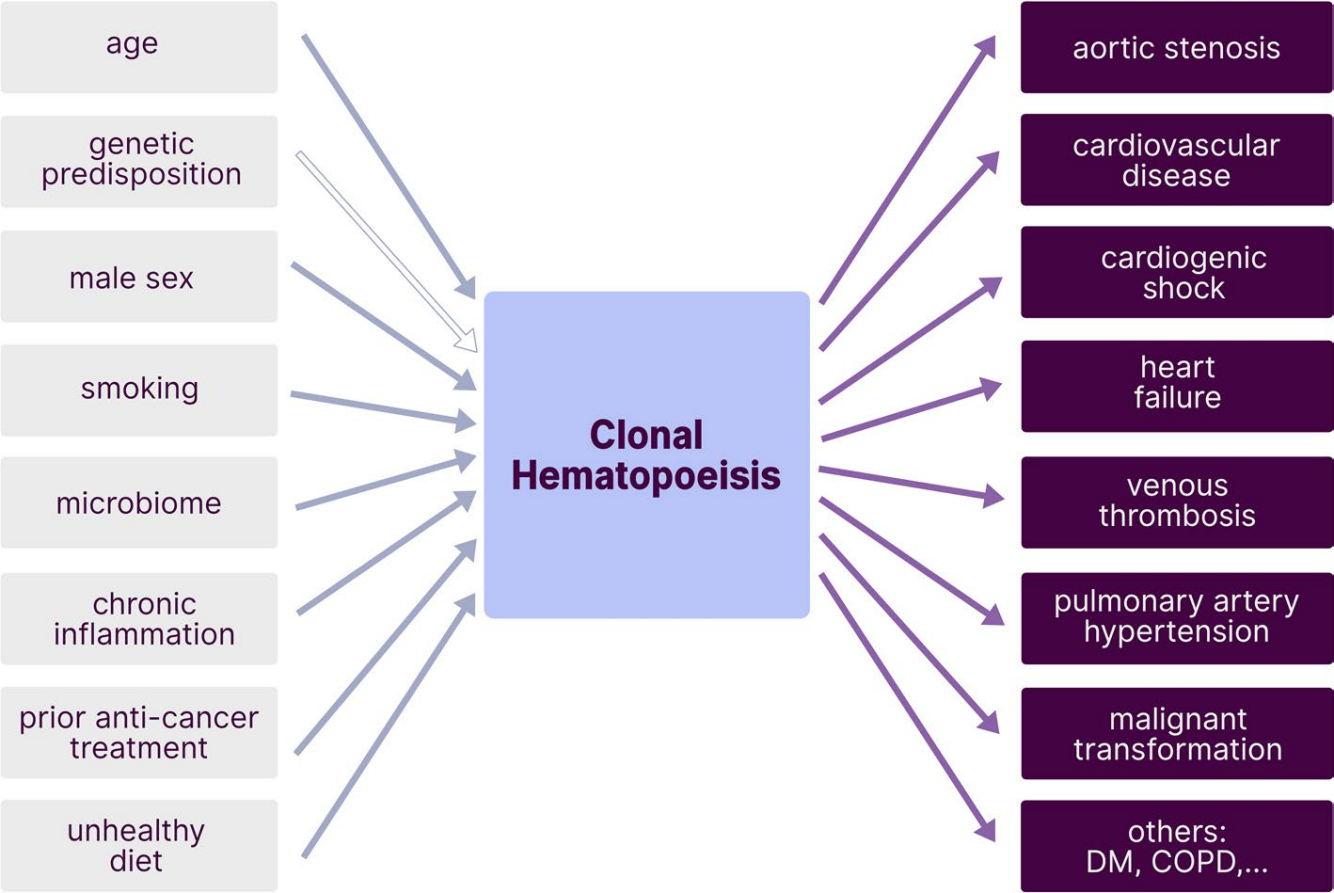
Risk factors and associated diseases of CH. Several circumstances (left) have been shown to promote the emergence of CH, with age representing the strongest risk factor. Regarding a genetic predisposition, current data remain contentious (empty arrow). However, CH is associated with an increasing number of diseases (right). Most of these are cardiac or malignant conditions. Nonetheless, associations with metabolic and chronic inflammatory disease have also been recently identified

The two most frequently affected genes are DNA (cytosine-5) methyltransferase 3 alpha (*DNMT3A*) and Tet methylcytosine dioxygenase 2 (*TET2*), followed by additional sex combs-like 1 (*ASXL1*) and serine/arginine-rich splicing factor 2 (*SRSF2*), Janus kinase 2 (*JAK2*) and Cbl proto-oncogene (*CBL*), among others [55]. These genes mostly encode for proteins involved in epigenetic regulation, such as DNA-methylation (*TET2, DNMT3A*) or histone modification (*ASXL1*), or for spliceosome constituents (*SRSF2, SF3B1*) or signaling proteins (*JAK2, CBL, GNB1, GNAS*) [8]. In contrast to established myeloid malignancies, such as acute myeloid leukemia (AML) or myelodysplastic syndromes (MDS), CHIP clones tend to be characterized by a more restricted range of mutations as well as a smaller clone size [8]. The predominant mutation pattern in CHIP is a single substitution of cytosine to thymidine, mostly due to spontaneous deamination. This is considered to be a signature of aging, as DNA error correction decreases in efficiency. It is therefore not surprising that age represents the strongest risk factor for CHIP [87]. By the age of 70, CHIP can be identified in 20–50% of all individuals depending on the sensitivity and gene coverage of the assay used to identify clonal variants [22, 46, 55]. Recent studies that use genome-wide approaches to detect clones that lack mutations in ‘known drivers’ of myeloid neoplasia, even suggest CH to be near-ubiquitous in the aging hematopoietic system, the reported prevalence of CH being limited only by the detection method used [5, 90, 94]. Besides age, some other factors predispose for developing CHIP (Fig. 1), such as previous oncologic treatment [16, 29, 50], male sex [43], smoking [55], unhealthy diet [11] and specific changes in microbiome composition [68]. Furthermore, chronic inflammatory diseases, such as auto-inflammation [7, 80, 96] or HIV-infection [33] seem to favor the emergence of CHIP (Fig. 1). The potential relevance of genetic predisposition remains contentious. Some studies point out a familial predisposition [48, 98] but this has not been supported by twin studies [20, 37, 44].

Although CHIP is associated with an increase in red cell distribution width [42], there is, by definition, no significant change in blood cell counts. This makes genetic analyses essential for screening and diagnosing CHIP. Since the reported prevalence of CHIP is strongly dependent on the sensitivity of the sequencing assay employed [53, 90], it is important to define the basic conditions for potential screening approaches.

Current definitions of CHIP, such as that adopted in the most recent 5^th^ edition of the WHO classification of hematolymphoid tumors, require detection of a variant in a known set of driver genes. However, it has recently become clear first, that expansion of clones with no such driver variants is common in the aging hematopoietic system [43, 69, 74, 98] and second, that CH without involvement of a known driver gene seems nonetheless to be associated with an increase in all-cause life-time mortality risk comparable to that of CHIP with a known driver [98].

## CHIP: a new cardiovascular risk factor

Independent of traditional cardiovascular risk factors, CHIP was shown to nearly double the cardiovascular risk compared to healthy individuals, thereby establishing CHIP as a new and persuasive cardiovascular risk factor. The individual risk increases with the size of the particular clone as represented by the VAF, which can vary between 2% (lower definition of CHIP) and 50% (equivalent to all cells carrying a heterozygous mutation) [42, 54, 55]. In the meantime, however, there is increasing evidence that CH clones well below 2% VAF can also impact cardiovascular life time [13, 54, 55].

Jaiswal et al. were the first to draw the connection of CHIP with CVD. Moreover, they provided a functional explanation. They exposed TET2-deficient and LDL-receptor defective mice, established by competitive bone marrow transplantation, to a high fat diet and observed a severe enlargement of atherosclerotic plaques [42, 54]. Similarly, an atherogenic diet of *Jak2*-mutant mice led to larger and more complex atherosclerotic plaques with larger necrotic cores, earlier lesion formation and greater pro-inflammatory immune cell activation compared to wild type mice [89].

Since the original identification of CHIP as a potent cardiovascular risk factor, several retrospective studies have further elucidated the connection between CHIP and common cardiovascular risk factors. For instance, CHIP occurs more frequently in smokers [16, 29, 31, 45, 49, 58]. Since tobacco is a mutagenic agent, the implication is clear. However, functional experiments identifying a direct causal effect are still lacking. There does seem to be a link between CHIP and metabolic imbalance, based on retrospective and in vitro data [18, 34, 41]. Cohort-analyses show that the presence of CHIP increases the likelihood of developing diabetes mellitus by 30% [18, 55]. Surprisingly, no associations were observed regarding lipid profiles, except for *JAK2*-mutations, which are negatively associated with total cholesterol and LDL cholesterol. Mechanistically, *TET2* is involved in regulation of the cellular metabolic homeostasis through AMP-activated protein kinase (AMPK), which represents a central cellular sensor of metabolic demand. In this context, hyperglycemia could promote a clonal advantage in *TET2* mutated stem cells [34]. In parallel, other groups have shown that depletion of *TET2* might accelerate atherosclerosis due to dysfunctional autophagy, as a major AMPK-regulated pathway [60, 97].

## Current evidence for clinical relevance of CHIP

### Atherosclerosis and myocardial infarction

In 2014 Jaiswal et al. were the first to describe a statistical association between atherosclerotic events and the presence of CHIP with a hazard ratio of 2.0 (95% confidence interval [CI] 1.1–1.8) for coronary artery disease (CAD) [55]. In a subsequent study, this group later compared 4726 CHIP-patients to a control group of 3529 individuals and confirmed a 1.9-fold overall risk of developing CAD for individuals affected by CHIP. They further measured the coronary artery calcification score based on computer tomography and detected a 3.3-fold increase in CHIP carriers. In line with this, the risk of developing early onset myocardial infarction, (age men < 40 years/woman < 50 years) was four times higher among CHIP-affected individuals. Closer analysis revealed two additional parameters to modulate the cardiovascular risk. First, risk was dependent on the clonal size (VAF). Second, it was dependent on the identity of the affected gene with mutations in *JAK2* leading to a 12-fold increase, whereas mutations in the most frequently affected genes *TET2* and *DNMT3A* conferred a two-fold increase in the risk of developing atherosclerosis [54]. The association between CHIP and CAD was replicated in a cohort of postmenopausal women where the likelihood was increased by a factor of 1.36 (95% CI 1.07–1.73) [49]. Since then, further clinical data have emerged from a range of CVDs (Table 1).

**Table 1:** Overview of current clinical data

Author, year	Cohort	Clinical data
Jaiswal et al. 2014 [55]	17,182 cases of 3 population-based cohorts	All-cause mortality (HR 1.4, 95% CI 1.1–1.8) Risk of coronary artery disease (HR 2.0, 95% CI 1.2–3.4) Ischemic stroke (HR 2.6, 95% CI 1.4–4.8)
Jaiswal et al. 2017 [54]	433 cases 577 controls of population-based cohorts	Cardiovascular risk (HR 1.9, 95% CI 1.4–2.7) Early onset myocardial infarction (HR 4.0, 95% CI 2.4–6.7)
Wolach et al. 2018 [91]	10, 893 cases 5947 healthy (232 CHIP) 4946 Schizophrenia (205 CHIP) (Institutional cohort of Dana-Farber Cancer Institute)	Doubled risk of venous thrombosis for all CHIP 12-fold increase for venous thrombosis for *JAK2*
Dorsheimer et al. 2019 [35]	200 cases with HF taken from trials examining the effects of intracoronary administration of autologous BMCs 38 cases with CHIP	4.4 years follow-up Higher mortality with HF hospitalization among *DNMT3A/TET2* (HR 2.1, 95% CI 1.1–4.0) Higher mortality associated w/ clonal size (VAF)
Abplanalp et al. 2020 [4]	8 cases with severe AS 6 postinfarction cHF 3 healthy controls	Sequencing monocytes of CHIP (*TET2* + *DNMT3A*) vs. non-CHIP. CHIP samples show increased expression of: IL-1b, IL-6 receptor, NLRP3 inflammasome complex, CD163
Assmus et al. 2020 [9]	419 cases of ischemic CHF 227 with *DNMT3A* or *TET2* (monocentric institutional)	Higher 5-year mortality among carriers of CHIP 5-year mortality without CHIP: 18% (95% CI 14–21%) 5-year mortality with one *DNMT3A* or *TET2*: 29% (95% CI 11–46%) 5-year mortality with both *DNMT3A* and *TET2*: 42% (95% CI 26–57%)
Bick et al. 2020 [13, 14]	97,691 cases 4229 with CHIP Community based cohort of NHLBI TOPMed research program	6.9 years follow-up Increase of CVD events among CHIP (HR 1.27, 95% CI 1.04–1.56) Greater risk of CVD from larger CHIP clone (HR 1.59, 95% CI 1.21–2.09)
Cremer et al. 2020 [30]	419 cases with HF (institutional cohort)	4-year follow-up Higher mortality among CHIP-carriers Higher mortality related to clonal size (VAF) Higher mortality related to mutation count
Mas-Peiro et al. 2020 [66]	279 cases with severe AS + TAVI 93 CHIP (*TET2/DNMT3A*) (monocentric institutional)	Increase in medium-term all-cause mortality following successful TAVI Adjustment for sex + age (HR 3.1, 95% CI 1.17–8.08) Adjustment for NT-proBNP (HR 4.81, 95% CI 1.49–15.57) No difference in clinical parameters
Potus et al. 2020 [75]	1832 PAH- patients 7509 controls 50 PAH-patients 41 healthy controls (PAH Biobank)	Identification of 9 unique *TET2*-germline variants 3 somatic variants 86% of PAH patients depicted reduced *TET2* expression
Bhattacharya et al. 2021 [12]	44 111 individuals 2507 with CHIP	10-years of follow-up The prevalence of CHIP increased among unhealthy diet (healthy 5.1%, intermediate 5.7%, unhealthy 7.1%) Increase of rates of incident cardiovascular events, compared to individuals without CHIP and intermediate diet to: Individuals with CHIP, unhealthy diet (HR 1.52; 95% CI 1.04–2.22) Individuals with CHIP, healthy diet (HR 0.99; 95% CI 0.62–1.58)
Honigberg et al. 2021 [49]	19,606 women 418 natural premature menopause 887 surgical premature menopause	Association with premature menopause (odds ratio, 1.36, 95% 1.10–1.68) CAD (HR 1.36, 95% CI 1.07–1.73) CAD among CHIP (VAF > 0.1) (HR 1.48, 95% CI 1.13–1.94)
Kiefer et al. 2021 [56]	399 cases with HF taken from clinical institutional trials	3.95 years follow-up Mutations within *CBL, CEBPA, EZH2, GNB1, PHF6, SMC1A, SRSF2* are associated with higher mortality independently of *TET2/DNMT3A*
Palomo et al. 2021 [70]	60 cases with HF 17 CHIP	3.6 years follow-up *DNMT3A* associated with diastolic dysfunction No increase of death among CHIP (HR 1.53, 95% CI 0.45–5.24) No increase of HF + death among CHIP (HR 2.12; 95% CI 0.79–5.71)
Pascual-Figal et al. 2021 [71]	62 HF cases 24 CHIP (single-center prospective registry of ambulatory patients)	3.65 years follow-up Accelerated HF progression among *DNMT3A/TET2* In terms of death (HR 2.79; 95% CI 1.31–5.92) Death or HF hospitalization (HR 3.84; 95% CI 1.84–8.04) HF-related death /HF hospitalization (HR 4.41; 95% CI 2.15–9.03)
Soudet et al. 2021 [82]	61 cases with unprovoked pulmonary embolism 12 CHIP (monocentric hospital of Amiens-Picardie)	No difference in terms of age, location or risk stratification
Yu et al. 2021 [95]	56,597 cases 3406 CHIP from 5 population-based cohorts, (ARIC, Atherosclerosis Risk In Communities, study; CHS, Cardiovascular Health Study; JHS, Jackson Heart Study; UKBB; WHI, Women’s Health Initiative	Increased prospective risk of HF (HR 1.25, 95%CI 1.13–1.38) *ASXL1, TET2 and JAK2* but not *DNMT3A* Higher risk for large CHIP-clones (HR 1.29, 95% CI 1.15–1.44) No difference with or without prior CAD
Bhattacharya et al. 2022 [11]	78,752 cases (8 prospective cohorts and biobanks)	Total stroke (HR 1.14, 95% CI 1.03–1.27) Hemorrhagic stroke (HR 1.24, 95% CI 1.01–1.51)
Böhme et. al. 2022 [15]	446 patients with cardiogenic shock after myocardial infarction (from CULPRIT-SHOCK randomized trial) 129 CHIP	Primary endpoint: 30-day all-cause mortality or renal replacement therapy Increased risk for combined endpoint (OR 1.83, 95% CI 1.05–3.21) Trend for difference in all-cause mortality (OR 1.67, 95% CI 0.96–2.90) after multivariable adjustment
Scolari et. al. 2022 [81]	341 patients with cardiogenic shock (mainly non-ischemic cause) vs. 345 patients with ambulatory heart failure	3-year follow-up Cardiogenic shock patients had a higher prevalence of CHIP (OR 1.5, 95% CI 1.0–2.1) Decreased survival among CHIP patients at different time points (30-days: HR 2.7; 95% CI 1.3–5.7; 90-days: HR 2.2; 95% CI 1.3–3.9; 3-years: HR 1.7; 95% CI 1.1–2.8)

Several prospective and retrospective cohort analyses draw correlations of CHIP with a wide range of cardiovascular diseases, such as heart failure, cardiovascular disease, aortic valvular stenosis, CVD and stroke

*AS* aortic stenosis, *BMCs* bone marrow cells, *CAD* coronary artery disease, *CHIP* Clonal hematopoiesis of indetermined potential, *CI* confidence interval, *HF* heart failure, *HR* hazard ratio, *cHF* chronic heart failure, *PAH* pulmonary artery hypertension

### Stroke

In line with the overall increased cardiovascular risk, the presence of a CHIP mutation was associated with a 2.6-fold increased likelihood for ischemic stroke in the cohort analyzed by Jaiswal et al. [55]. This increased risk of total strokes was replicated in a further cohort, following adjustment for age, sex and ethnicity, with a hazard ratio of 1.14 (95% CI 1.03–1.2). Importantly, the risk for hemorrhagic stroke was increased 1.24-fold, with *TET2* being the strongest risk associated gene. Overall, CHIP-bearers were prone to hemorrhagic rather than to ischemic stroke. Among ischemic strokes, *TET2* again showed the strongest association, with microvascular pathology being more frequent than large-vessel strokes [11].

### Heart failure

The outcomes of patients with heart failure (HF) has repeatedly been associated with CHIP [9, 30, 35, 56, 70, 71], with correlations resembling those in atherosclerosis. Namely, the HF risk increases with clone VAF [9, 35, 56, 95] and with the number of the acquired mutations [30].

Atherosclerosis represents the major cause of congestive HF [12] and CHIP-carriers confer a significantly increased HF-associated mortality after having experienced myocardial infarction [30, 35]. Inflammation appears to lay an important role here [61]. In mouse models lacking TET2 or DNMT3A, blockage of Interleukin-1β (IL-1b) rescued the deficiency in heart function [78, 79]. Furthermore, transcriptome analysis of HF patients with *DNMT3A* mutations reveal elevated expression levels of inflammatory genes [4]. Hence, one possibility might be that CHIP is associated with HF through inflammation-mediated effects on atherosclerosis. Nevertheless, in the cohort of Dorsheimer et al., most of the deaths among *TET2* or *DNMT3A*-mutation carriers were attributed to arrhythmic events or progression of HF rather than to myocardial infarction [35]. Furthermore, a large meta-analysis including over 55,000 participants failed to identify any difference between CHIP carrying HF patients regarding prior CAD [95]. Sano et al. investigated the pathogenesis of HF upon CHIP-mutations by establishing a mouse model system. For this purpose, they infused angiotensin II to mimic hypertensive HF and observed increased cardiac dysfunction associated with lack of TET2 or of DNMT3A. Interestingly, the phenotype induced by loss of TET2 or DNMT3A was accompanied by increased cytokine release and expansion of the hematopoietic compartment [79].

Cardiogenic shock represents the most severe clinical manifestation of acute heart failure. Böhme et al. recently studied the prognostic impact of CHIP in 446 patients with cardiogenic shock in acute myocardial infarction from the CULPRIT-SHOCK randomized clinical trial [15]. CHIP variants at ≥ 2% VAF were found in 29% of the patient population, most frequently in the *DNMT3A* and *TET2* genes. Compared to non-CHIP patients, CHIP carriers experienced worse short-term clinical outcome with regard to a composite endpoint of all-cause mortality and the need for renal replacement therapy, even after adjustment for traditional risk factors and several established biomarkers such as arterial lactate, NT pro brain natriuretic peptide (NT-proBNP), cystatin C or IL-6. In line with this, Scolari et al. observed a 1.5-fold increased prevalence of CHIP-mutations, particularly *TET2* and *ASXL1*, among patients with cardiogenic shock predominantly of non-ischemic origin, compared to HF patients (95% CI 1.0–2.1). At the same time, harboring a CHIP mutation was associated with a decrease of survival at multiple timepoints (30-days: HR 2.7; 95% CI 1.3–5.7, *P* = 0.006; 90-days: HR 2.2; 95% CI 1.3–3.9, *P* = 0.003; and 3-years: HR 1.7; 95% CI 1.1–2.8, *P* = 0.01), while individuals carrying a *TET2*-mutation displayed elevated serum levels of SCD40L, IFNγ, IL-4 and TNFα [81].

### Aortic stenosis and other conditions

Sequencing a cohort of 279 patients with aortic valve stenosis (AS) having undergone transcatheter aortic valve intervention (TAVI) identified another association with CHIP. Mortality between one and eight months after TAVI was increased for carriers of *DNMT3A* or *TET2* by a factor of 3.1 (95% CI 1.17–8.08) after adjustment for sex and age, and by 4.8-fold (95% CI 1.49–15.57) after further adjustment for NT-proBNP serum levels. Interestingly, basic clinical parameters such as concomitant atherosclerotic disease, blood cell count, inflammatory markers or procedural characteristics were comparable between CHIP and non-CHIP patients [66]. Another study analyzed monocytes of AS patients carrying CHIP-mutations and detected an increased expression of inflammatory genes and mediators, including IL-1b, IL-6-receptor, NLRP3 inflammasome complex and CD163 [4].

Compared to the other common CHIP mutations, those in *JAK2* carry a specific risk profile. Wolach et al. identified a 12-fold increased risk for venous thrombosis among *JAK2-*mutation carriers, compared to a doubled risk among the other CHIP mutations. The authors offered mechanistical insight using *Jak2*^V617F^ knockin mouse model, in which increased formation of neutrophilic extracellular traps, as a mechanism of innate immunity, promoted thrombosis [91]. However, an independent study analyzed a cohort of 61 patients with unprovoked pulmonary embolism, 12 of whom carried CHIP-mutations, and failed to detect any difference between CHIP carriers and non-CHIP carriers. Possibly due to the small number of CHIP-positive individuals, no *JAK2* mutation was found [82].

Another association with CHIP, and in particular with *TET2* mutation*,* was recently demonstrated for pulmonary arterial hypertension (PAH) within a cohort of 1832 individuals. In this case, contradictory to the aforementioned data, the identification of a *TET2* mutation predicted a favorable course of disease, with disease onset being delayed and pulmonary artery pressure being lower among *TET2* mutation carriers. Nonetheless, the group found in accompanying mouse experiments that depletion of *Tet2* did provoke PAH and this effect was reversible by blockage of IL-1b [75].

## Mechanisms of increased CV risk in persons with CH

### Macrophage activity and cytokine release

**Fig. 2:**
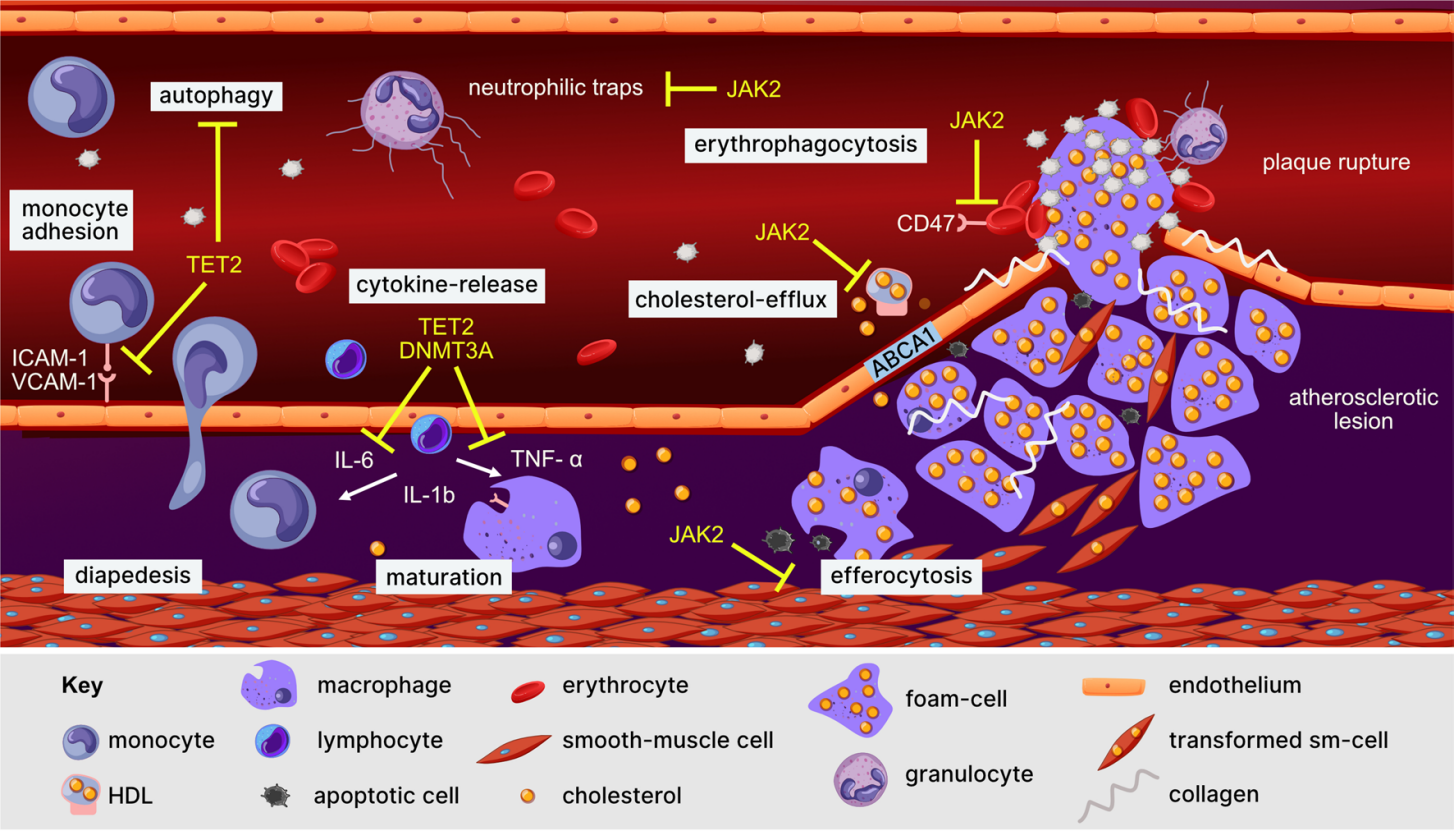
Functional interconnections between CHIP and CVD. Somatic mutations within CHIP-associated genes contribute to atherosclerotic pathomechanisms at various stages. Possible interferences of CHIP and atherogenesis are depicted in yellow. Effects appear gene-specific. Notably, some mutations may lead to loss (as assumed for most *TET2*-mutations or the hotspot-mutation R822H within *DNMT3A*) and others to gain of function, which raises a special challenge in the search for common mechanisms of CHIP-associated risk increases. *sm* smooth muscle cell, *ICAM-1* Intercellular Adhesion Molecule 1, *VCAM-1* vascular cell adhesion protein 1, *ABCA* ATP-binding cassette transporter A1

Atherosclerosis represents a severe vascular pathology, driven by the interplay between dyslipidemia and inflammation (Fig. 2). Given the evidence discussed above, it seems likely that CHIP promotes a pro-inflammatory environment, including changes of monocyte and macrophage biology, implying that chronic inflammation might embody the portentous link between cardiovascular risk and CHIP.

This conclusion was based initially on the observation of significantly increased cytokine levels in association with CHIP mutations. A direct causal association between CHIP and atherosclerosis via inflammation was subsequently demonstrated by Fuster et al. [41], who observed increased levels of IL-1b and an apparent atherosclerotic phenotype in *Tet2* mutant mice. Blockage of IL-1b diminished the arteriosclerotic phenotype, identifying inflammation as the crucial connection [42]. Amelioration of atherosclerosis and HF in *Tet2* depleted mice by pharmacological inhibition of the NLRP3 inflammasome (MCC950), followed by reduction of IL-1b further confirmed this functional concept [79].

These cytokines strongly regulate monocyte rolling, adhesion and transmigration through endothelium, followed by macrophage transformation, all of which are key processes contributing to atherosclerosis [59]. Global changes in cytokine levels and skewed monocytic and macrophagic functions therefore represent a compelling elicitor for the vascular pathology.

The majority of research in this area has analyzed the effects of variants in the two genes most frequently mutated in CH: *TET2* and *DNMT3A*. As described above, *TET2*-depletion resulted in increased release of the cytokines IL-1b, IL-6 and tumor necrosis factor-alpha (TNF-α) in vitro [1, 42, 51, 78], as well as in mouse models [42]. Similar observations were made for *DNMT3A*. For instance, CRISPR/Cas-9 mediated *DNMT3A* knockdown provoked elevated levels of the cytokines CXCL1, CXCL2, IL-6 and CCL5 in macrophages [78]. Furthermore, monocytes bearing *DNMT3A* mutations showed an increased expression of pro-inflammatory genes, including the aforementioned cytokines [3, 14, 61]. In line with these in vitro data, CHIP-carriers also displayed elevated serum levels of IL-6 [14, 28], IL-8 [54], and IL1-b [14].

While modified global cytokine levels apparently represent a major hallmark in terms of CHIP mediated pathomechanism, there also appear to be changes in specific monocytic and macrophagic functions, although this effect seems to be context specific [27]. For instance, patients with germline variants of *DNMT3A*, showed reduced levels of monocyte secreted IL-10 [67]. In another experiment, *DNMT3A* stimulated antiviral immune response of macrophages through activating histone deacetylase 9 [64]. In contrast, monocyte-function appeared to remain mainly stable in the presence of *TET2* mutations [56], with the exception of the promotion of macrophage migration inhibitory factor (MIF), a pivotal factor for monocytic differentiation, that is characteristically elevated in atherosclerosis [76]. With respect to *Jak2*-mutants, alterations in macrophage function also appear to contribute to atherosclerosis [89]. Here, it has been shown experimentally that *Jak2* mutant macrophages induce DNA replication stress, activate the AIM2 inflammasome and thereby aggravated atherosclerosis [39].

Taking the pro-inflammatory state in atherosclerosis into account, it is not surprising that elevated levels of high-sensitive C-reactive protein (hs-CRP), a non-specific indicator of inflammation, are observed [59]. However, with regards to CHIP contradictory data exist. Some studies found no association of CHIP and hs-CRP [14, 59], whereas others state a significant elevation [21]. Altogether, CHIP appears to disturb the strongly cytokine regulated equilibrium of macrophage expansion and function, which further promotes the development of atherosclerotic lesions.

### Lipoprotein- and cellular metabolism

Lipoprotein homeostasis is a further factor contributing to the formation of atherosclerosis. Macrophages that arise from transmigrated circulating monocytes are able to ingest oxidized or native LDL inside the atherosclerotic lesion. If this process is dysfunctional or macrophages become overloaded, they can turn into foam cells and contribute to arteriosclerotic lesion progression [59]. Just recently, CHIP was found to have an impact in this process. Dotan et al. investigated mutant *Jak2* mice and macrophages. They observed attenuated cholesterol efflux from macrophages, most likely due to dysfunctional ABCA1 cholesterol transporter, while cholesterol uptake was unaffected, leading to the acceleration of atherosclerosis. Consistent with this, systemic inhibition of *JAK2* with ruxolitinib provoked the same effect [36]. In large CHIP cohorts, however, no alterations of serum cholesterol and its derivates were detected [14, 36, 54, 55].

One of the major hubs to sense and regulate cellular energy and substrate supply is the ubiquitous and highly conserved process of autophagy. Macroautophagic degradation is thought to accelerate atherosclerosis at several breaking points, such as efferocytosis, lipid metabolism and oxidative homeostasis of the endothelium [62]. THP1-derived macrophages, that were incubated with Ox-LDL, showed an attenuation of their macrophagic activity in a *TET2* dependent manner, and effect that was reversible by mimicking de-methylation through treatment with azacytidine [60]. Immunohistochemical staining of aortic walls in mice revealed both reduced expression of *Tet2* and reduced autophagic activity upon low shear stress. Changes in the expression of *TET2* were shown to regulate autophagic activity in endothelial cell cultures, with the knockdown of *Tet2* being accompanied by decreased expression of endothelial NO-synthase and increased expression of endothelin-1 as a typical hallmark of endothelial dysfunction [93]. Where it is of note that a recent report of endothelial cell differentiation form circulating monocytoid cells in a sheep model raises the possibility that CHIP myeloid cells may have the potential to transdifferentiate and contribute directly to the endothelial compartment [11]. In ApoE^−/−^ mice, overexpression of *Tet2* was followed by upregulation of autophagy reduced atherosclerotic lesions and a decrease in the expression of ICAM-1 and VCAM-1, which are crucial for monocyte invasion. Despite this, cholesterol, tri-acyl-glyceride, and lipoprotein levels remained unchanged [72]. Together, these data indicate an involvement of TET2 in the regulation of autophagy in macrophages and endothelial cells. A potential mediator in this respect is Beclin-1, which is central to the assembly of autophagosomes and the expression of which is controlled by TET2-dependent de-methylation of promotor sequences [72]. It is worth noting in this context that changes in DNA methylation-patterns have widely been associated with increased atherosclerotic risk [6, 63, 73].

In the case of *JAK2,* further mechanisms have been discussed. As described above, since *JAK2* mutations provoke neutrophils to form neutrophil extracellular traps, this may lead to thrombosis, which could further increase CVD risk [91]. Furthermore, *Jak2*^*V617F*^ mice showed increased atherosclerotic lesions with complex and comparatively large necrotic cores containing iron, erythrocyte and macrophagic depositions. Reduced erythrocyte expression of CD47 and reduced levels of c-Mer tyrosine kinase (MerTK), as a key modulator of efferocytosis, suggest that impaired efferocytosis and increased erythrophagocytosis may make substantial contributions to the formation of atherosclerotic lesions [89].

## Cardio-oncology axis: future perspectives

CHIP mediates a novel and unique bi-directional connection between hematopoiesis and the cardiovascular system, in a functional as well as clinical manner. The exact mechanisms by which some mutations promote a clonal advantage and accelerate atherosclerosis, for instance by provoking inflammation, remain to be elucidated. From what is known so far, CHIP mutations convey enhanced self-renewal of the hematopoietic stem cell (HSC) compartment and concomitantly obstructed hematopoietic differentiation in a mutation-specific fashion [24, 25, 47]. The most frequent mutations within *TET2* and *DNMT3A* further promote granulomonocytic differentiation to the expense of the erythroid lineage [57, 88]. These effects seem to be mediated by the epigenetic enzymatic functions, which coordinate the action of transcription factors regulating self-renewal or myeloid lineage commitment [24]. Notably, the enhancement of HSC-proliferation itself may accelerate CH-development [47]. The currently adopted concept hypothesizes a vicious cycle between enhanced inflammation triggered by CHIP and the emergence of more mutations [52].

Similar mutual associations exist regarding the likelihood of malignant transformation and the prevalence of CHIP among oncologic patients. On the one hand, CHIP carriers bear a higher likelihood of malignant transformation [2, 32, 43], on the other hand radio and chemotherapy increase mutation rate and reduce the stem cell pool, thus increasing proliferative pressure on the remaining cells. This is likely to contribute to the higher prevalence of CHIP among pre-treated oncology patients [29, 65, 92]. Given the strong association of CHIP with increase of cardiovascular life-time risk and the fact that some hematological diseases such as MDS are associated with increased cardiovascular risk anyway [19], this cohort clearly deserves thorough observation or follow-up. With respect to screening approaches, it may be appropriate to routinely subject oncological patients to close surveillance of both cardiovascular function and CHIP mutation status. The presence of a CHIP mutation may ultimately impact treatment decisions.

## Potential therapeutic approaches to mitigate CH-associated CV risk

Because of the far-reaching clinical implications of the entity CHIP, there is clearly a case for surveillance and potential preventive intervention. Given the mutual connections between CHIP, malignant disease and CVD, a closely intertwined interdisciplinary communication is likely to be essential for effective clinical management. Having recognized this necessity, some clinics already offer specific CHIP consultation [17, 83]. However, no standard guidance procedures or general recommendations regarding the adequate counseling of affected patients have been established to date. Nonetheless, expert recommendations include a tight monitoring of traditional cardiovascular risk factors, individual risk assessment, adjustment of lifestyle factors and notification of the whole care team. Regarding interventions with the potential of curbing cardiovascular risk, no evidence-based options are available yet, beyond changes in lifestyle and common risk factors. For instance, current data suggests an impact of nutrition, since screening of 48,289 individuals unraveled a higher prevalence of CHIP among those consuming an unhealthy diet, defined as a decreased ratio between healthy elements (fruit and vegetables) to unhealthy elements (red meat, processed food and added salt) [10].

If a clonal somatic mutation is detected in a patient with unexplained cytopenia, further diagnostic testing may be indicated to rule out an underlying overt hematological malignancy. In the event of a dynamic change in blood parameters, a bone marrow puncture should be performed [17].

Nevertheless, screening is only clinically justified if there are potential interventions or pre-emptive treatment options. Initial in vitro experiments have already identified first pharmacological targets (Table 2). One aims to restore TET2’s enzymatic activity by supplementing its cofactor ascorbic acid, which was shown to reverse the enhanced self-renewal of hematopoietic stem and progenitor cells (HSPC) in *Tet2*-deficient mice [26]. An ongoing clinical trial addresses this relationship by investigating the effect of intravenous high dose ascorbic acid in patients bearing CCUS (NCT03418038), while other clinical approaches target inflammatory processes. Another promising mode of action was examined as part of the CANTOS-trial (Canakinumab Anti-inflammatory Thrombosis Outcome Study). Herein a sub-study observed the effects of the anti-IL-1b antibody Canakinumab on individuals with CHIP [77, 86]. First results indicate a significant reduction of cardiovascular events in patients bearing *TET2*-mutations [85]. Some trials targeting mutated isocitrate dehydrogenase (IDH) by Enasidenib (NCT05102370) and Ivosidenib (NCT05030441) respectively, are under recruitment. In *ASXL*-knockin-mice, activation of autophagic activity by blockage of mTOR through rapamycin reversed the phenotype of HSPC expansion [8, 40] thereby identifying a potential future pharmacological point of application.

**Table 2:** Current ongoing clinical trials on diagnosis and treatment of CHIP

Clinical trial	Condition	Intervention	Identifier
STOP-LEUKEMIA: Repurposing Metformin as a Leukemia-preventive Drug in CCUS and LR-MDS	CCUS, LR-MDS	Metformin	NCT04741945
A Pilot Study of Enasidenib for Patients with Clonal Cytopenia of Undetermined Significance and Mutations in IDH2	CCUS	Enasidenib	NCT05102370
A Pilot Study of Ivosidenib for Patients with Clonal Cytopenia of Undetermined Significance and Mutations in IDH1	CCUS	Ivosidenib	NCT05030441
Metabolic Profiling of Hematopoietic Stem Cells in Clonal Hematopoiesis (CHIP): A Prospective Observational Study	CHIP	Prospective Single-cell transcriptomics Mutation-specific single-cell genotyping	NCT05246813
Clonal Hematopoiesis of Indeterminate Potential and Residual Cardiovascular Event Tendency After Smoking Cessation	CHIP	Prospective (1-year) whole-exome sequencing	NCT04987268
Impact of Donor Clonal Hematopoiesis of Indeterminate Potential (CHIP) on Recipient Outcome Following Allogeneic Hematopoietic Stem Cell Transplantation (Allo-HSCT)	CHIP	Prospective nanopore long-read sequencing	NCT04689750
Screening of Clonal Hematopoiesis of Indeterminate Potential in Venous Thromboembolism	CHIP	Retrospective	NCT04477564
Phase 2 Trial of High Dose Intravenous Ascorbic Acid as an Adjunct to Salvage Chemotherapy in Relapsed/Refractory Lymphoma and Patients with Clonal Cytopenia of Undetermined Significance	CHIP	High dose intravenous ascorbic acid	NCT03418038
Is Clonal Hematopoiesis of Indeterminate Potential Associated with Unprovoked Pulmonary Embolism?	CHIP	DNA-sequencing	NCT04711746
A Single centre cohort study to determine if clonal hematopoiesis of indeterminate potential (CHIP) is a risk factor for chemotherapy-related complications in lymphoma patients ≥ 60 of age receiving cytotoxic chemotherapy	CHIP	DNA-sequencing	NCT04053439

In conclusion, CH represents a promising field for future containment of cardiovascular risk as well as hematological malignancies. As a prerequisite, further interconnections besides promotion of an inflammatory milieu (such as metabolic and mesenchymal interplay) remain to be elucidated and are likely to reveal further targets for potential pre-emptive treatment options. With respect to HF, the crucial question of the ways in which CHIP, inflammation and atherosclerosis are intertwined in disease evolution and affect prognosis demands further attention. With this goal, this review aims to increase the awareness for CH and point out the areas most relevant for future research. A more thorough appreciation of the diverse interactions and mechanisms can be expected to identify fresh opportunities for translation into the clinic not just for the treatment, but increasingly for the prevention of CVD.

## Abbreviations

AS: Aortic stenosis
AML: Acute myeloid leukemia
CAD: Coronary artery disease
CCUS: Clonal cytopenia of undetermined significance
CHIP: Clonal hematopoiesis of indetermined potential
CVD: Cardiovascular disease
DNMT3A: DNA-methyl-transferase 3A
IL: Interleukin
JAK2: Janus kinase 2
MDS: Myelodysplastic syndrome
MI: Myocardial infarction
PAH: Pulmonary artery hypertension
TAVI: Transcatheter aortic valve intervention
TNF: Tumor necrosis factor
VAF: Variant allele frequency
